# Improving the Quality of Systematic Reviews in Spinal Surgery Requires Community-Wide Engagement and Pragmatism

**DOI:** 10.1177/2192568220952758

**Published:** 2020-11-04

**Authors:** Ben Grodzinski, Oliver Mowforth, Lindsay A. Tetreault, Benjamin M. Davies

**Affiliations:** 1 12204 University of Cambridge, Cambridge, UK; 2 5894 New York University Langone, New York, NY, USA

To the Editor:

We read with interest the study by Dettori et al,^1^ which concludes that there is “critically low confidence” among spinal surgery systematic reviews (SRs). Their conclusions are drawn using the AMSTAR 2 tool for SRs published in 2018 from 4 leading spine journals (N = 28).

This is a compelling headline, and while we welcome the spotlight on SR methodology, we urge caution when interpreting these results. First, the AMSTAR is considered by many a checklist of reporting rather than an absolute assessment of methodological quality.^2^ Second, each point on the AMSTAR 2 is given equal weighting, which may not reflect their individual significance. For example, the use of 2 reviewers for data extraction has not been shown to substantially affect treatment effect estimates,^3^ but receives equal weighting to the reporting of conflicts of interest, highly relevant to spinal surgical evidence.^1,4^ Finally, the reproducibility of this tool is unclear, with a degree of subjectivity to several points.^2^

Regardless of the flaws of the AMSTAR 2, SRs have a very important role in supporting evidence-based medicine and represent an increasing proportion of published spinal literature.^5^ Given this, it is undoubtedly important to ensure surgeons can have confidence in their findings.

While Dettori et al^1^ highlight the challenges faced by study authors in writing a spinal surgery SR, perhaps a bigger challenge is those factors outside the control of individual researchers.

For example, in the field of Degenerative Cervical Myelopathy (DCM), there is inconsistent disease terminology, and an absence of index terms or codes. The result is that reliable study identification can only be done manually, with a consequent element of subjectivity.^6^ Second, and perhaps more significant, outcome reporting in spinal surgery is extremely heterogeneous, with many different aspects of the disease measured, using different tools and at different time points. The result is that SRs often restrict their inclusion criteria to a specific tool. While this will often enable quantitative “meta-analysis,” it does so at the expense of excluding relevant literature and could introduce bias. This can even afflict studies rated as “high confidence” by AMSTAR 2. Alternatively, more inclusive approaches to SR can be used, as outlined in the recent Synthesis Without Meta-analysis (SWiM) reporting guidelines. However, this method prevents quantitative analysis such as the pooling of effect sizes.

Addressing these fundamental issues however will require a longer term and community-wide commitment. Within spinal surgery, recommendations on outcome reporting have been established for Deformity Surgery (COSSCO) and Cauda Equina Syndrome (CESCOS), while our own process for DCM (AO Spine RECODE-DCM) is ongoing.^7^ The impact of these recommendations will take time and are contingent on their adoption.

We thank Dettori et al^1^ for placing a spotlight on SRs in spinal surgery, and advocate for community-wide solutions to increase confidence in spinal surgery SRs.
